# Examining the Concordance of Detection of Hereditary Cancer Gene Variants Between Blood, Tumour, and Normal Tissue in Patients with High-Grade Serous Ovarian Carcinoma

**DOI:** 10.3390/genes16111260

**Published:** 2025-10-25

**Authors:** L. Mui, J. Kerkhof, C. M. McLachlin, K. Panabaker, J. McGee, B. Sadikovic, E. A. Goebel

**Affiliations:** 1 Department of Pathology and Laboratory Medicine, Schulich School of Medicine and Dentistry, Western University, London, ON N6A 5C1, Canada; 2 Verspeeten Clinical Genome Centre, London Health Sciences Centre, London, ON N6A 5W9, Canada; 3 Department of Pathology and Laboratory Medicine, London Health Sciences Centre, London, ON N6A 5A5, Canada; 4 Department of Pediatrics, Medical Genetics, London Health Sciences Centre, London, ON N6A 5W9, Canada; 5 Department of Obstetrics and Gynecology, London Health Sciences Centre and Schulich School of Medicine and Dentistry, Western University, London, ON N6A 5W9, Canada

**Keywords:** ovarian carcinoma, BRCA, molecular testing, hereditary cancer panel, germline testing, next generation sequencing

## Abstract

Background/Objectives: Access to genetic counselling and BRCA1/2 germline testing is standard of care for patients with high-grade serous ovarian carcinoma (HGSOC). While tumour testing reliably detects pathogenic variants in hereditary cancer genes, it cannot distinguish somatic from germline variants. Concurrent testing of non-cancerous (normal) tissue obtained during surgery may improve triage for germline testing and clinical genetics referral. This study evaluated the concordance of inherited variant detection among tumour, normal tissue, and blood to determine whether archived normal tissue can reliably identify germline pathogenic variants. Methods: Patients with HGSOC who had a pathogenic variant identified by targeted Next Generation Sequencing (NGS) tumour testing and underwent germline hereditary cancer gene panel (HCP) testing between April 2019 and November 2020 were included. HCP testing was performed on formalin-fixed, paraffin-embedded normal tissue from the original resection. Variant results were compared across tumour, normal tissue, and germline (blood) samples to determine concordance, false-negative, and false-positive rates. Results: Forty-one patients had confirmed tumour variants in BRCA1/2 or other HCP genes. Of these, 24 harboured a corresponding germline pathogenic variant. Archived normal tissue was available for 23 of these 24 cases, and all germline variants were detected in normal tissue, showing 100% concordance. Among the 17 patients without germline variants, all corresponding normal tissue samples were negative, also demonstrating 100% concordance. No false positives or negatives were identified. Conclusions: NGS testing of normal tissue at surgical resection reliably identifies germline pathogenic variants in patients with HGSOC. Incorporating this approach may help triage patients for clinical genetics assessment.

## 1. Introduction

Genetic counselling and germline testing for *BRCA1/2* is considered standard of care for all patients diagnosed with high-grade serous ovarian carcinoma (HGSOC) [1]. Germline testing can identify patients with hereditary variants, trigger downstream genetic testing of family members, and prompt specific cancer-prevention strategies in affected individuals [1,2]. In current Canadian practice, every patient diagnosed with HGSOC is recommended to undergo germline testing and genetic counselling [3]. In the past, an opt-out referral process for genetic consultation has been implemented to better facilitate pathways to genetic testing of patients, maximize identification of hereditary variants, and trigger preventative care strategies. Though this process is feasible, effective, and patient-centered, there is a relative shortage of genetic counsellors and specialists in Canada compared to the number of eligible patients [3,4]. As a result, this strategy, designed to capture as many eligible patients as possible for genetics consultation, meets a bottleneck due to prolonged wait times, which may negatively affect patient care. Additionally, this strategy inevitably captures a subset of patients with HGSOC who do not have germline variants and may not require genetic consultation [3].

Currently, a “mainstreaming” approach is used, where germline testing is a means of prioritizing or triaging patients for Clinical Genetics consultation by confirming the presence of germline variants prior to proceeding with the referral. However, it requires an additional phlebotomy procedure and can introduce a new waiting period for patients, as germline test results can take months to return, depending on the test [5]. Timely reporting of test results is a critical component of patient-centered care. Longer testing wait times result in treatment delays, contributing to patient harm [5]. The introduction of tumour testing is a feasible and reliable method of detecting somatic and germline pathogenic variants in hereditary cancer genes. Tumour testing of HGSOC resection specimens can detect *BRCA1/2* Tier I/II and American College of Medical Genetics (ACMG1/2) variants reliably while identifying somatic variants independent of germline status [6,7]. As such, reflex testing of tumour specimens at the time of surgical resection can be used as a strategy to improve patient triage for germline testing and referral to clinical genetics. Testing performed using targeted Next Generation Sequencing (NGS) panels has a relatively quick turnaround time and can be routinely reported as part of, or supplemental to, the routine pathology report completed on each resection specimen. However, tumour testing alone cannot readily distinguish somatic variants from germline variants and only confirms the presence of a variant within a known cancer gene in the specimen [7]. The objective of this study is to examine the concordance of detection of hereditary cancer gene variants between blood, tumour, and normal tissue in patients with HGSOC using a custom clinical HCP of 37 genes. We aim to demonstrate the feasibility of normal tissue testing to reliably capture and identify pathogenic germline variants.

## 2. Materials and Methods

### 2.1. Study Population

The study population included a retrospective cohort of all patients with HGSOC at our institution from April 2019 to November 2020, in which a pathogenic variant was identified in their tumour tissue by targeted NGS. Somatic (tumour tissue) testing was previously performed by NGS, using a clinically validated 37-gene HCP, which enabled the simultaneous detection of both sequence (SNV) and copy-number variants (CNV). Germline (blood) testing was previously performed using clinically validated assays considered the gold standard at the time of original testing (NGS, Sanger, MLPA, or DHPLC).

### 2.2. Sample Preparation and Testing

Histological slides from the patient’s original cancer surgical resection specimens were examined for normal, non-carcinoma-involved tissue. In cases where sufficient normal tissue was identified (cervix, fallopian tube, lymph node, ovary, adnexal soft tissue, small bowel, colon, or uterus), archived formalin-fixed, paraffin-embedded normal (non-carcinoma involved) tissue from a block containing no tumour was retrieved and tested using NGS to identify possible pathogenic variants in hereditary cancer genes. The results were then compared to the variant profile of the patient’s prior somatic and germline testing.

### 2.3. Hereditary Cancer Panel

The previously clinically validated 37-gene HCP examines all coding exons and 20 bp of flanking intronic sequence of 37 genes (*APC*, *ATM*, *BARD1*, *BMPR1A*, *BRCA1*, *BRCA2*, *BRIP1*, *CDH1*, *CDK4*, *CDKN2A*, *CHEK2*, *CTNNA1*, *EPCAM*, *FANCC*, *FANCM*, *FLCN*, *GREM1, HOXB13*, *MEN1*, *MLH1*, *MSH2*, *MSH3*, *MSH6*, *MUTYH*, *NBN*, *NTHL1*, *PALB2*, *PMS2*, *POLD1*, *POLE*, *PTEN*, *RAD51C*, *RAD51D*, *SDHB*, *SMAD4*, *STK11*, *TP53*). *TP53* is included in the HCP but not assessed for the purposes of this study. Sensitivity detection of this custom NGS pipeline has been validated previously for the minor allele detection levels of 2–5%, along with sub-exon level CNV detection [8].

DNA isolation, NGS, and detection of copy-number variants were described previously [7]. In brief, 20 μm sections from formalin-fixed paraffin-embedded (FFPE) tissue were obtained for DNA isolation. NGS libraries were prepared with 100 ng of genomic DNA in a pre-capture pool of 24 specimens, and the captured pool was diluted to 1.3 pM for sequencing on an Illumina NextSeq instrument [9,10]. Sequence variants were filtered at an allelic fraction of >10% to minimize the impact of sequence artifacts and to focus on germline inheritance, and CNVs were identified through a quantile normalization algorithm with a sensitivity threshold of 30% [9,10]. Variants were classified by a clinical molecular geneticist based on the American College of Medical Genetics and Genomics (ACMG) standards and guidelines for pathogenicity [11,12]. For this study, all assessed ACMG 1/2 variants (pathogenic or likely pathogenic) [12] were reviewed for concordance with the previously assessed tumour and blood tissues.

### 2.4. Data Analysis

Results are reported using descriptive statistics. For samples in which normal tissue testing, tumour testing, and matched germline testing were available, variant identification was compared to determine concordance. Concordance rate, false-negative, and false-positive rates were calculated.

## 3. Results

**Figure 1:**
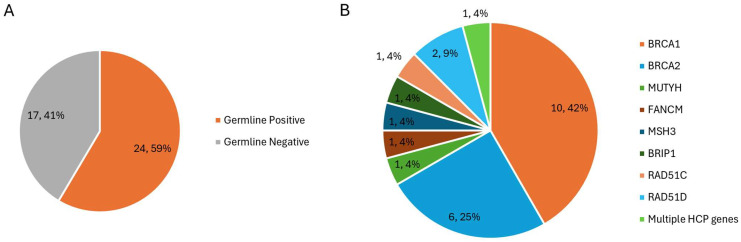
Distribution of germline testing results for the 41 patients identified with tumours positive for a pathogenic variant in a Hereditary Cancer Panel (HCP) gene. The proportion of patients with HGSOC with an identified germline variant is shown in (**A**). The distribution of affected genes within the germline variant group is shown in (**B**).

Forty-one patients with HGSOC and a confirmed pathogenic variant in *BRCA1/2* or other HCP gene on tumour testing were identified from the study period. Of these 41 cases, 24 (56%) had a previously identified matching germline variant from peripheral blood testing. Of the 24 cases with germline variants, 16 patients had a germline pathogenic or likely pathogenic variant in *BRCA1* (*n* = 10) or *BRCA2* (*n* = 6), and the remaining 8 had one or multiple pathogenic or likely pathogenic variants in other HCP genes: *MUTYH*, *FANCM*, *MSH3*, *BRIP1*, *RAD51C*, and *RAD51D* (Figure 1).

The remaining 17 patients with a confirmed pathogenic variant on tumour testing did not have the matching variant identified by germline testing, indicating a likely somatic variant isolated within the tumour specimen.

In total, 40 of the 41 cases had sufficient normal, non-carcinoma-involved tissue available for NGS testing. In one case, sufficient normal, non-carcinoma-involved tissue was not available for testing. The results of normal tissue testing were compared to the previously analyzed germline (blood) and somatic (tumour) results (Table 1).

**Figure 2:**
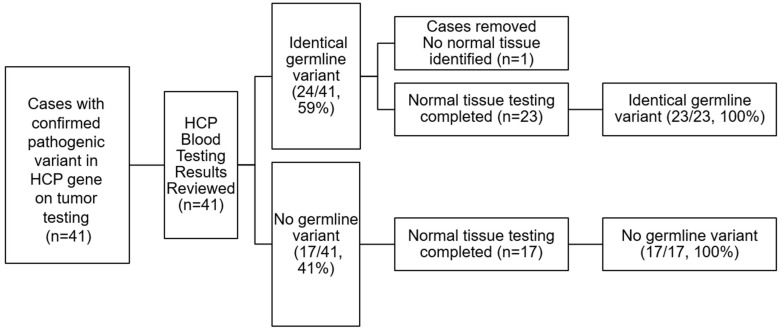
Summary of HGSC tumour, matched HCP germline testing, and normal tissue testing results. After cases with a pathogenic variant in the HCP gene on tumour testing were identified and germline results were reviewed, cases were separated into two groups: 1. Tumour cases with an identical germline variant identified, and 2. Tumour cases with no germline variant identified. Normal tissue testing was performed on all cases where normal tissue was present. There was 100% concordance between germline testing and normal tissue testing, with 100% of germline positive cases having identical normal tissue testing results, and 100% of germline negative cases having identical negative normal tissue testing results.

In the 23 cases included with a confirmed pathogenic germline variant, NGS testing of normal tissue identified the same variant. Tumour testing also showed an identical variant in all 23 cases, with 2 cases revealing an additional pathogenic gene variant in the tumour. No pathogenic variants were identified in the normal tissue of the remaining 17 cases with a pathogenic variant identified in the tumour, but no germline variant was identified (Figure 2). There was 100% concordance between germline HCP testing and normal tissue testing. No false positive or false negative results were identified.

## 4. Discussion

Germline testing for hereditary variants, including *BRCA1/2*, has traditionally been performed on peripheral blood specimens with Sanger sequencing [13]. With the advent and continuing development of NGS and other molecular testing methodologies, genetic testing turnaround time and efficiency have greatly improved. Testing of formalin-fixed paraffin-embedded (FFPE) tissues for genetic variants is also now achievable, offering a powerful alternative to the previously established standard [14]. Multiple studies have assessed the feasibility of variant testing with NGS in FFPE tumour tissues [15], including in cases of prostate [16,17], breast [17,18], and ovarian cancers [6,17,19]. These studies have found that tumour testing with NGS can reliably identify variants of interest. Additionally, several studies have looked at the feasibility of testing archived FFPE non-cancerous normal tissues for germline variants by comparing their performance with that of fresh liquid, blood, or tissue biopsy germline testing [18,20,21,22,23,24,25]. These studies have reported high concordance in results, despite known artefacts caused by formalin fixation [22,23,24,26].

Our study corroborates these findings and demonstrates that NGS testing normal tissue within a resection specimen in patients with HGSOC can reliably detect hereditary germline variants, showing 100% concordance with standard-of-care blood-based hereditary cancer panel testing. Various studies have directly compared the genetic testing results of blood and FFPE normal tissue testing in patients with specific cancers, including breast [21], ovarian [27], prostate [28], and hepatocellular cancers [29]. Concordance between the results in those studies has been observed to be high [23]. Additionally, our study results have found that normal tissue testing can achieve this concordance without inadvertently capturing patients who have somatic tumour mutations only. This means that concurrent tumour and normal tissue testing may be able to select patients who may not need a genetic counselling consultation for their HGSOC, further streamlining the consultation pipeline and better allocating resources in a limited resource environment.

In accordance with our results and other results in similar studies, DNA from non-cancerous FFPE tissues in cancer patients can serve as an alternative and reliable source for the assessment of germline variants. If normal tissue testing were to be performed reflexively as part of routine pathology assessment at the time of diagnosis, this could have implications for hereditary gene variant detection, as this could potentially identify tumour variants which are likely to be of germline origin. If reflex testing is rapid and completed before a patient’s post-operative follow-up appointment, there may be a role for reflex testing in triaging genetic referrals for patients with HGSOC. In Canada, genetic counselling is available to all patients with HGSOC; however, HCP germline test turnaround time is variable, wait times for a genetics consultation are often high, and referral rates can be low on a center-to-center basis [4,30]. We, therefore, propose an alternative pipeline to identify patients for genetics referral by testing a patient’s normal tissue if their tumour testing confirms a pathogenic or likely pathogenic variant. In our study, up to 41% of individuals with tumour variants identified on NGS had no variant detected in normal tissue and, therefore, could have been deprioritized for genetics consultation, allowing other patients with likely germline variants to be triaged for consultation appropriately and expediently. However, a negative genetic test in the normal tissue sample may not preclude consideration of a referral for genetic counselling and further testing if other risk factors, such as significant family history of cancer, are present.

Normal tissue testing also opens alternative avenues for downstream familial testing in cases where patients diagnosed with HGSOC may not have had traditional germline (blood) testing. In such patients who are now deceased, testing of their normal tissue would allow for identification of heritable variants, providing surviving family members with this valuable information. A validation study on genetic testing of archival FFPE normal tissues in patients with known *BRCA1/2* pathogenic variants found a high success rate in DNA extraction and high concordance with germline blood testing results in calling pathogenic variants of *BRCA1/2* specifically [6,31]. Our results are consistent with these findings but also support that this concordance rate holds true for other hereditary variants in addition to *BRCA1/2* in HGSOC. This could be significant in healthcare frameworks that do not trigger downstream testing of family members without a confirmed positive hereditary variant identified by germline testing, and allow families to access genetics counselling and improved hereditary risk assessment beyond evaluation of family history alone. The Canadian Association of Pathologists recommends keeping paraffin blocks and slides for surgical pathology for up to 20 years, though limitations in storage and maintenance vary by institution, with most retaining tissue storage for at least 10 years [32]. This means the opportunity to pursue this line of testing would exist for even years after the passing of the affected patient and allow family members the time to process the diagnosis and the passing of their relative and consider the benefits or drawbacks of pursuing such testing without feeling pressured by an immediate deadline on when the opportunity expires.

Limitations to this study include that this is a retrospective study of a 41-patient cohort, and further prospective studies in a larger patient cohort would provide greater insight into the utility of normal tissue testing results in triaging patients for genetics consultation. A second limitation for consideration is the potential burden that FFPE NGS testing of normal tissue imposes on the molecular laboratory. FFPE specimens can be more technically challenging to process than blood and could represent an increased work burden on the lab, albeit continuing improvements in automated tumour tissue sampling and DNA extraction, as well as FFPE sequencing and analysis technologies, are making this less of a challenge. However, with the proposed triage testing pipeline, it is predicted that fewer referrals to genetics and fewer germline blood tests will be necessary, thereby potentially negating the additional burden of increased FFPE testing.

Since there were no false negative variants in tumour NGS testing, considerations for increasing the efficiency of testing normal tissue could include further refinement of testing methods, such as consideration of targeted Sanger testing instead of a broad NGS panel for FFPE normal tissue after tumour NGS testing. Targeted Sanger testing may offer a faster and less expensive testing option in these cases.

Finally, tissue samples in this study consisted primarily of archived specimens less than 14 years old. Formalin fixation of tissue is known to damage DNA and introduce artefacts [33], and the amount of amplifiable DNA in paraffin blocks has been shown to decrease with specimen age [34]. Further research with a wider specimen pool, including older specimens and using newer molecular testing technologies, will be needed to determine the limits of tissue age and performance of molecular technologies. Identification and selection of normal, non-cancerous tissue should be carefully considered to avoid any cross-contamination with tumour tissue. In our study, normal tissue was selected from a separately submitted section containing no tumour. In one case, normal tissue was not identified in the surgical specimen, and, therefore, testing could not be performed. It is important to ensure only normal tissue is included for testing, particularly if non-cancerous tissue is in close proximity to the tumour, in order to avoid false positive results.

## 5. Conclusions

This study finds that NGS testing of normal tissue using a 37-gene hereditary cancer panel, including *BRCA1/2*, shows 100% concordance with known germline findings in cases of HGSOC. These results suggest that reflex testing of normal tissue may be a viable method for germline variant detection in this patient cohort, supporting a more patient-centered and timely triage process for genetic counselling. Further, prospective studies with larger cohorts are needed to evaluate the impact and broader clinical utility of this approach.

## Figures and Tables

**Table 1:** Summary of tumour variants identified in each case, with corresponding variant detection results in blood and normal tissue testing.

Patient	Reportable Variant Detected in Tumour	Variant Detected in Blood	Variant Detected in Normal Tissue
1	NM_007294.3(BRCA1):c.(4185+21_4186-21)_(4357+21_4358-21)del (50%)	Yes	Yes
2	NM_000059.3(BRCA2):c.7069_7070delCT, p.(Leu2357Valfs*2) (18.7%)	Yes	Yes
3	NM_000059.3(BRCA2):c.3545_3546delTT, p.(Phe1182*) (34%)	Yes	Yes
4	NM_007294.3(BRCA1):c.4327C>T, p.(Arg1443*) (46.1%)	Yes	Yes
5	NM_007294.3(BRCA1):c.3748G>T, p.(Glu1250*) (52%)	Yes	Yes
6	NM_000059.3(BRCA2):c.7988A>T, p.(Glu2663Val) (55.7%)	Yes	Yes
7	NM_007294.3(BRCA1):c.2071delA, p.(Arg691Aspfs*10) (66.5%)	Yes	Yes
8	NM_000059.3(BRCA2):c.5303_5304delTT, p.(Leu1768Argfs*5) (76%)	Yes	Yes
9	NM_007294.3(BRCA1):c.4689C>G, p.(Tyr1563*) (88.1%)	Yes	Yes
10	NM_007294.3(BRCA1):c.427G>T, p.(Glu143*) (96.3%)	Yes	Yes
11	NM_007294.3(BRCA1):c.3254_3255dupGA, p.(Leu1086Aspfs*2) (57.6%)	Yes	Yes
12	NM_000059.3(BRCA2):c.7958T>C, p.(Leu2653Pro) (99.4%)	Yes	Yes
13	NM_007294.3(BRCA1):c.5266dupC, p.(Gln1756Profs*74) (58.4%)	Yes	Yes
14	NM_000059.3(BRCA2):c.3170_3174del, p.(Lys1057Thrfs*8) (71.4%)	Yes	Yes
15	NM_007294.3(BRCA1):c.68_69delAG, p.(Glu23Valfs*17) (76.3%)	Yes	Yes
16	NM_000059.3(BRCA2):c.(?_-21)_(*21_?)del (40%)	Yes—MUTYH only	Yes—MUTYH only
	NM_001128425.1(MUTYH):c.1187G>A, p.(Gly396Asp) (57%)		
17	NM_007294.3(BRCA1):c.(?_-21)_(*21_?)del (35%)	Yes—FANCM only	Yes—FANCM only
	NM_020937.2(FANCM):c.5101C>T, p.(Gln1701*) (52.4%)		
18	NM_000059.3(BRCA2):c.8164dupA, p.(Thr2722Asnfs*8) (37.3%)	Yes—MUTYH and RAD51D only	Yes—MUTYH and RAD51D only
	NM_001128425.1(MUTYH):c.1187G>A, p.(Gly396Asp) (64.4%)		
	NM_002878.3(RAD51D):c.620C>T, p.(Ser207Leu) (61%)		
19	NM_000314.4(PTEN):c.741dup, p.(Pro248Thrfs*5) (33.5%)	Yes—MSH3 only	Yes—MSH3 only
	NM_002439.4(MSH3):c.703C>T, p.(Gln235*) (36.2%)		
20	NM_032043.2(BRIP1):c.2255_2256del, p.(Lys752Argfs*12) (57.6%)	Yes	Yes
21	NM_058216.1(RAD51C):c.224dup, p.(Tyr75*) (77.1%)	Yes	Yes
22	NM_002878.3(RAD51D):c.668-2A>C (82.1%)	Yes	Yes
23	NM_000077.4(CDKN2A):c.341C>A, p.(Pro114His) (26.6%)	Yes—RAD51D only	Yes—RAD51D only
	NM_002878.3(RAD51D):c.649_655delinsTGAGGTT, p.(Gly217*) (65%)		
24	NM_007294.3(BRCA1):c.212+3A>G (96.7%)	Yes	No Result
25	NM_007294.3(BRCA1):c.(?_-21)_(*21_?)del (25%)	No	No
26	NM_000059.3(BRCA2):c.(?_-21)_(*21_?)del (35%)	No	No
27	NM_007294.3(BRCA1):c.(?_-21)_(*21_?)del (50%)	No	No
28	NM_000059.3(BRCA2):c.(?_-21)_(*21_?)del (50%)	No	No
29	NM_007294.3(BRCA1):c.(?_-21)_(*21_?)del (55%)	No	No
30	NM_000059.3(BRCA2):c.(?_-21)_(*21_?)del (40%)	No	No
31	NM_000059.3(BRCA2):c.(?_-21)_(*21_?)del (50%)	No	No
32	NM_007294.3(BRCA1):c.3569delC, p.(Pro1190Leufs*20) (11.4%)	No	No
33	NM_007294.3(BRCA1):c.3339T>A, p.(Tyr1113*) (35%)	No	No
34	NM_000059.3(BRCA2):c.7758G>A, p.(Trp2586*) (43.2%)	No	No
35	NM_007294.3(BRCA1):c.1961dupA, p.(Tyr655Valfs*18) (33.9%)	No	No
36	NM_007294.3(BRCA1):c.4986+3G>T (69.3%)	No	No
37	NM_007294.3(BRCA1):c.2603C>A, p.(Ser868*) (90.5%)	No	No
38	NM_007294.3(BRCA1):c.1912G>T, p.(Glu638*) (65.1%)	No	No
	NM_000059.3(BRCA2):c.(?_-21)_(*21_?)del (30%)		
39	NM_007294.3(BRCA1):c.3394_3406del, p.(Asn1132Leufs*19) (70%)	No	No
40	NM_007294.3(BRCA1):c.2891delG, p.(Gly964Aspfs*36) (58.4%)	No	No
41	NM_000314.4(PTEN):c.1004G>A, p.(Arg335Gln) (17%)	No	No

## Data Availability

This study received ethics approval from the Office of Human Research Ethics at Western University and the Lawson Health Research Institute (REB#115598), with approval granted on 26 April 2021.
